# Barbell reveals and resolves demultiplexing and trimming issues in Nanopore data

**DOI:** 10.1093/bioinformatics/btag349

**Published:** 2026-06-02

**Authors:** Rick Beeloo, Ragnar Groot Koerkamp, Xiu Jia, Marian J Broekhuizen-Stins, Lieke van IJken, Els M Broens, Aldert Zomer, Bas E Dutilh

**Affiliations:** Department of Biology, Science4Life, Utrecht University, Utrecht, 3584 CH, The Netherlands; Department of Computer Science, Karlsruhe Institute of Technology, Karlsruhe, 76131, Germany; Institute of Biodiversity, Ecology, and Evolution, Faculty of Biological Sciences, Cluster of Excellence Balance of the Microverse, Friedrich Schiller University Jena, Jena, 07743, Germany; Division of Infectious Diseases and Immunology, Utrecht University, Utrecht, 3508 TD, The Netherlands; Division of Infectious Diseases and Immunology, Utrecht University, Utrecht, 3508 TD, The Netherlands; Division of Infectious Diseases and Immunology, Utrecht University, Utrecht, 3508 TD, The Netherlands; Division of Infectious Diseases and Immunology, Utrecht University, Utrecht, 3508 TD, The Netherlands; Department of Biology, Science4Life, Utrecht University, Utrecht, 3584 CH, The Netherlands; Institute of Biodiversity, Ecology, and Evolution, Faculty of Biological Sciences, Cluster of Excellence Balance of the Microverse, Friedrich Schiller University Jena, Jena, 07743, Germany

## Abstract

**Motivation:**

Oxford Nanopore sequencing enables long-read analysis for diverse applications, but artefacts introduced by Nanopore barcoding are poorly characterized and can compromise demultiplexing accuracy and downstream analyses.

**Results:**

Using a rapid barcoding experiment on 66 diagnostic samples, we found that only 83% of reads followed the expected single-barcode configuration, while 17% showed complex barcode attachments. We observed similar patterns in public datasets, and also in native barcoding datasets where only 30%–70% of the reads had barcodes on both ends. Widely used demultiplexers, including Dorado, fail to resolve these cases, leaving ∼10% of our rapid barcoding reads partially trimmed and contaminated with adapter fragments. We developed Barbell, a pattern-aware demultiplexer that is designed to detect complex barcode configurations. Barbell reduced contaminated reads from >400 000 (Dorado/Flexiplex) to 166 (99.96% reduction), minimized barcode bleeding, and supports custom experimental designs such as dual-end barcodes and shorter barcodes (e.g. Illumina barcodes). We further show that such contamination is widespread in public databases, with Nanopore sequences detected in hundreds of NCBI entries, some of which are responsible for artificial taxonomic connections.

**Availability and implementation:**

Barbell is open source and available at https://github.com/rickbeeloo/barbell.

## 1 Background

Nanopore sequencing offers real-time, long-read DNA and RNA sequencing with minimal capital investment and laboratory footprint. The R10.4.1 pore architecture with its dual-head design and improved basecalling models increased sequencing accuracy beyond 99% (Kim *et al.* 2024), enabling applications in 16S amplicon sequencing (Karst *et al.* 2021), genome assembly (Wick *et al.* 2023), and metagenomic analysis (Charalampous *et al.* 2019).

To increase throughput and reduce cost, Nanopore experiments commonly multiplex samples using molecular barcodes introduced during library preparation via tagmentation, ligation, or PCR. In PCR-based barcoding, barcode sequences are incorporated into primer 5${\;}^{\prime }$ ends, tagging each amplicon without additional ligation steps. In tagmentation, transposase fragments DNA while inserting barcoded adapters at cut sites. In ligation, barcoded adapters are enzymatically joined to intact DNA fragment ends. After sequencing, reads are assigned to their source samples through demultiplexing, which involves identifying barcode sequences and often trimming them from read termini.

Despite improved sequencing accuracy, demultiplexing remains error-prone. Barcode bleeding (sample cross-talk or leakage) occurs at rates of 0.056%–1.5% (Xu *et al.* 2018, Wu *et al.* 2021, Sauvage *et al.* 2023, Holzschuh *et al.* 2024), primarily from concatenated reads or low-confidence barcode sequences (Xu *et al.* 2018). Incomplete adapter trimming further complicates downstream analyses (Bonenfant *et al.* 2023, Liu-Wei *et al.* 2024), particularly for low-abundance targets or diagnostic applications (Wu *et al.* 2021, Holzschuh *et al.* 2024).

Current demultiplexers face key challenges: Dorado lacks support for custom configurations (see https://github.com/nanoporetech/dorado/issues/626), Splitcode cannot practically handle high indel rates (Sullivan and Pachter 2024) (Error patterns up to a defined edit distance are precomputed and stored in a hash map. For long sequences or large edit thresholds, the combinatorial space of possible patterns becomes intractably large.), and Flexiplex supports only single-end barcodes (Cheng *et al.* 2024). Moreover, none report abnormal barcode attachment patterns, and all assume perfect ligations/tagmentations. These limitations reduce accuracy and restrict custom protocol adoption.

### 1.1 Contributions

We introduce Barbell, an extensive tool for demultiplexing that contributes on several fronts:

- Insight into experimental errors and barcode attachment patterns in Nanopore sequencing.
- Handling of complicated custom experimental set-ups [e.g. multiple primers, shorter/longer barcodes (e.g. Illumina barcodes), and dual-end barcodes)].
- New barcode scoring scheme reflecting Nanopore errors.
- The option to only include reads displaying safe tagmentation patterns or maximize assignment (e.g. for assembly).

We compared Barbell with existing demultiplexers Dorado and Flexiplex. Our evaluation included trimming errors and the effects of read contamination on taxonomic assignment and genome assembly. We also assessed contamination in NCBI “core nt”, overall showing that:

- Only ≈80% of our rapid barcoding reads contain the expected single barcode on one side of the read, with the remainder having more complex configurations.
-
≈10% of trimmed reads by Dorado and Flexiplex still contain experimental contamination, whereas this is near 0 for Barbell.
- Contamination in trimmed reads propagates into assemblies and produces artificial taxonomic assignments.
- Many sequences in NCBI’s database contain Nanopore contamination, including assemblies and amplicons.

## 2 Materials and methods

### 2.1 Preliminaries

In this manuscript, we address the problem of demultiplexing, where the goal is to locate a *tag*, denoted by τ, of length |τ|, within a read $R=r_{0}\ldots r_{n-1}$ of length n:=|R|. Both τ and *R* are strings over the alphabet {A,C,G,T}, extended with IUPAC ambiguity codes (e.g. R,Y,M,N). Let σ:=|Σ| denote the alphabet size.

Each tag τ consists of three parts (or substrings): a left flank $F_{\ell }$, a barcode *B*, and a right flank $F_{r}$. We denote their respective lengths as $\left|F_{\ell }\right|$, |*B*|, and $\left|F_{r}\right|$, such that $\tau :=F_{\ell }{}^{\circ}B{}^{\circ}F_{r}$, where ° denotes string concatenation. The barcode *B*, typically 24 bp, comes from a set of *g* known barcodes, $\beta =\{b_{1},b_{2},\ldots ,b_{g}\}$, whereas $F_{\ell }$ and $F_{r}$ are fixed strings with lengths varying based on the protocol, from $|F_{r}|=8$ for native barcoding kits, to, e.g. $|F_{r}|=50$ for rapid barcoding.

Since all barcodes share the same flanks, we first locate $F_{\ell }$ and $F_{r}$, and restrict the barcode search to the region between them. We replace *B* with wildcards (*N*) to obtain $\tau_{N}:=F_{\ell }{}^{\circ}N_{\left|B\right|}{}^{\circ}F_{r}$, and align this pattern to the read. The aligned *N*-region defines the substring between the flanks. Due to indels that optimize flank alignment, this substring may be shorter or longer than |*B*|. It is then compared against the barcode set β.

### 2.2 Scoring/penalizing methods

Throughout the manuscript we use two ways of penalizing or scoring sequences. The first measure is the edit distance, which we use to locate $\tau_{N}$ in the read. The second is a subsequence-based scoring function that we use to discriminate between barcodes. This second score is more sensitive to the localized error patterns typical of Nanopore sequencing. Briefly, the subsequence score is derived from the CIGAR string of an edit-distance–based alignment and is designed to reward long, contiguous runs of exact matches. Alignments dominated by uninterrupted matches receive high scores, whereas interleaved patterns of matches and errors receive low scores. A formal definition of the score, together with its motivation and implementation details, is given in Appendix B, available as supplementary data at *Bioinformatics* online.

#### 2.2.1 Cut-offs for flank and barcode matching

To locate the flanks ($\tau_{N}$), Barbell applies automatic cut-offs to distinguish true matches from random alignments. These cut-offs are derived from alignment scores observed in random sequence data of the same effective length, using an empirically fitted lower bound (Fig. 3, available as supplementary data at *Bioinformatics* online). For barcode assignment, subsequence scores are normalized relative to the maximum achievable (perfect-match) score, and barcodes are accepted based on user-defined minimum score and score-separation thresholds. A full derivation of these cut-offs and their empirical motivation is provided in Appendix C, available as supplementary data at *Bioinformatics* online.

### 2.3 Demultiplexing

Barbell has four main steps: annotate, inspect, filter, and trim.

#### 2.3.1 Annotate

**Figure 1:**
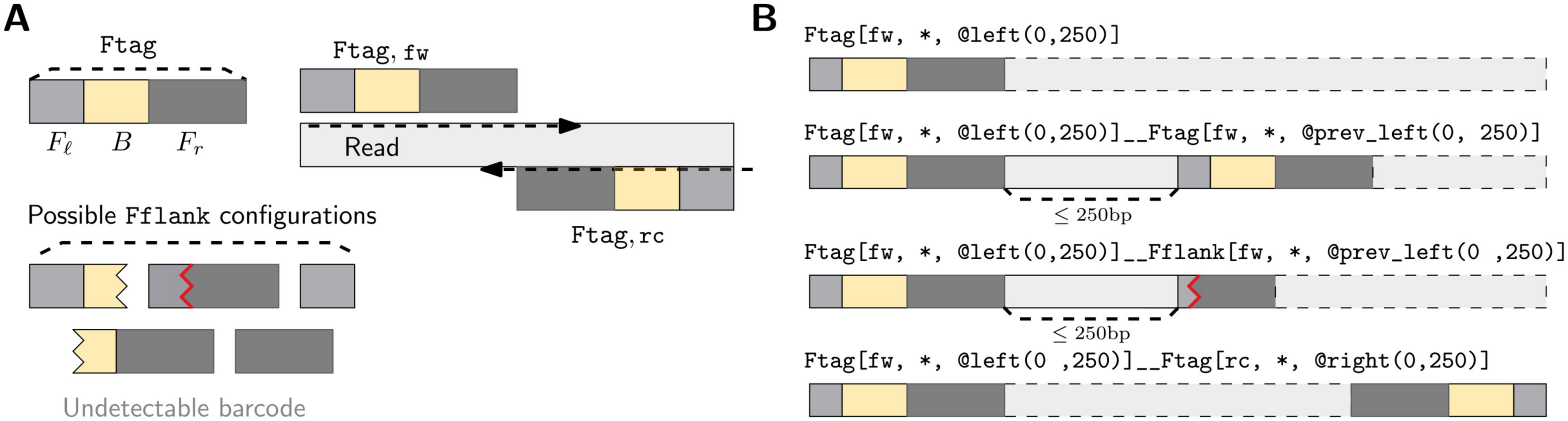
Example patterns observed in rapid barcoding data. (A) shows that an Ftag consists of the left flank ($F_{\ell }$), the barcode (*B*), and the right flank ($F_{r}$). If *B* is undetectable based on the scoring scheme (e.g. absent or bad score) we report it as Fflank. If an Ftag or Fflank matches the user-provided sequence, it is reported as fw; if it matches in reverse complement, as rc. In (B) there are several examples of match patterns observed in rapid barcoding data. The dashed borders (--) indicate the part of the read retained after trimming when using the Barbell rapid barcoding maximize preset. By default, a “grouping” of 250 bp is used, as tags are generally shorter than this; however, this value can be modified as a parameter in inspect.

**Figure 2:**
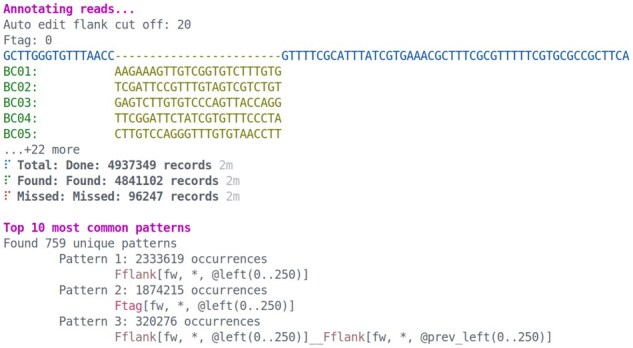
Barbell command-line interface. Example output when running barbell kit -kit SQK-RBK114-24 -i reads.fastq -o output. The interface displays kit information, including whether the --maximize option was used (Section 2.3). Inferred flanks (blue), detected barcodes (yellow), and the automatically assigned flank edit-distance cutoff (20 in this example) are shown. The output reports progress at each step and summarizes the most frequent sequence patterns in the input Fastq file (3/10 shown), providing a direct overview of double attachments and other experimental artefacts.

In the annotate step we locate and score barcodes/flanks in the reads, producing a tsv file with matches per read. In this manuscript, we focus on rapid barcoding, but all Nanopore kits are supported (e.g. SQK-NBD114.96). The algorithm is described in Algorithm 1, available as supplementary data at *Bioinformatics* online. In short, the user supplies a Fasta file (or multiple Fasta files) containing the tag sequences, from which Barbell derives $\tau_{N}$ and β. Barbell then locates $\tau_{N}$ in the reads, extracts the masked region, and compares it to each barcode in β. Whether a barcode matches is based on the subsequence score. By default, the score for *b* should be ≥20% of the perfect score, and the difference between the top two should be ≥10%. To get an intuition of these parameters and their effect on the number of demultiplexed reads, see Fig. 2, available as supplementary data at *Bioinformatics* online. If a barcode is found, this is reported as Ftag, where the F denotes front, otherwise the flank is reported as Fflank (Fig. 1A). In case of dual-end barcodes, the user can provide an additional Fasta file with an Rtag (R for rear), of which the incomplete Rtag is reported as Rflank. For all its searches, Barbell uses Sassy with a default overhang penalty α=0.5, which halves the edit cost for bases that align beyond the read boundary. This allows detection of truncated tags at read ends (Beeloo and Groot Koerkamp 2026a).

#### 2.3.2 Pattern representation

The annotate step produces per-read “matches”, which Barbell translates into patterns used by both inspect and filter. A read pattern consists of one or multiple matches, each represented as <type>[<ori>, <label>, <pos>, <cut>], where <type> denotes the match class (e.g. Ftag, Rtag, Fflank, Rflank), <ori> the strand orientation (fw or rc), <label> the barcode label derived from the Fasta header, <pos> a location constraint relative to the read end: @left(0.0.250) and @right(0.0.250), or relative to the previous match: @prev_left(0.0.250). Here 0.0.250 indicates a range of 0 to 250 bases and can be tuned to match the expected distances. The <cut> indicates the trim direction used in trim (≫ keep after, ≪ keep before). These match representations are joined to form the patterns depicted in Fig. 1B.

#### 2.3.3 Inspect

After assigning a pattern to each read we can count how often patterns are observed to provide an overview of barcode locations and multi-barcode ligation events (Fig. 2; bottom).

#### 2.3.4 Filter

In the filter step, reads are matched to a pattern and optional trim rules (Fig. 1B). These patterns can be copied from the inspect overview or manually refined, e.g. to keep only a specific label. The user can also specify where the read should be cut; for instance, Ftag[fw, *, @left(0.0.250), ≫] trims the Ftag from the left and keeps the sequence to the right. By default, Barbell only accepts the unambiguous ligation patterns for each kit, but - -maximize relaxes this filter to retain additional patterns, such as reads with different barcodes on both ends, at the cost of lower assignment confidence. Reads that pass the filter, together with their trim coordinates, are written to a filtered tsv file; trimming itself is performed in the trim step.

#### 2.3.5 Pattern ambiguity

Inspect and filter serve different purposes. Inspect shows all patterns detected in the reads, whereas filter can be used to extract a subset of reads matching a certain pattern. Here, patterns are not necessarily unambiguous. For example a *tag* may match both @left(0.0.250) and @right(0.0.250) in the case of very short reads. Similarly, a *tag* might be close to the previous tag [@prev_left(0.0.250)] and the right end [@right(0.0.250)]. Inspect always prioritizes grouping based on @prev_left(i.j) over @right(i.j).

#### 2.3.6 Trim

The trim step uses the tsv file from filter to trim the reads. For example, Ftag[fw, *, @left(0.0.250), ≫] retains the read section after the tag match, and Ftag[fw, *, @right(0.0.250), ≪] retains the read section before the tag match. Here, 0.0.250 only specifies that the tag lies within the first 250 bases; trimming itself is performed based on the exact alignment position of the tag. Tags can be combined for dual-end barcoded reads, e.g. Ftag[fw,*,@left(0.0.250),≫]__Rtag[≪,rc,*,@right(0.0.2 50)] which trims both ends extracting the region between the barcodes (Fig. 1B; dashed lines). This step creates an output folder with trimmed reads.

### 2.4 Further methods

In Appendix A, available as supplementary data at *Bioinformatics* online, we provide further details on the DNA extraction of the experimental isolates, the genome assemblies, and contamination searching.

### 2.5 Barbell tool: usage and applications in custom experiments

Barbell is available as a command-line tool, and for standard Nanopore kits (e.g. SQK-RBK114-96), a single command automatically identifies flanks and barcodes, sets cut-offs, performs trimming, and generates summary statistics (Fig. 2). The tool further accommodates custom experimental designs, including dual-end barcodes and mixed amplicon datasets by allowing users to define their own flanking sequences and barcodes.

## 3 Results

We developed Barbell to demultiplex Nanopore reads. To obtain experimental data for testing we first performed a Nanopore rapid barcoding experiment (SQK-RBK110.96) where we sequenced 66 diagnostic samples (BC01 to BC66). Then we explored barcode contamination in public data, and how it affects downstream analyses.

### 3.1 Demultiplexed reads

Sequencing of the 66 diagnostic samples yielded a total of 4 937 349 reads which we demultiplexed with Dorado, Flexiplex, and Barbell. Dorado assigned 4 647 221 (94.1%) (Dorado unexpectedly retained reads consisting entirely of barcode sequence; we excluded 52 778 such reads for a fair comparison, but users should be aware of this behaviour.) to a barcode, Flexiplex 4 667 336 (94.5%), and Barbell 4 224 099 (85.6%). For Barbell we used the - -maximize option (Section 2.3), and - -use-extended which detects *fused* barcodes (discussed later in Section 3.2).

The number of demultiplexed reads is a quantitative measure, not necessarily a qualitative one, as we explore in the next sections. The average runtimes were 6 min 50 s for Dorado, 1 min 2 s for Flexiplex, and 4 min 20 s for Barbell. Throughout the following sections we often refer to “patterns” as described in Section 2.3 and Appendix A.1, available as supplementary data at *Bioinformatics* online.

### 3.2 Patterns in rapid barcoding data

#### 3.2.1 Common patterns in reads

Rapid barcoding is designed to attach a single barcode to one end of each read, and in our dataset we observed this expected pattern in 83.0% of reads (4 095 862; Table 1). In total, 680 distinct barcode attachment patterns were detected: 5.3% (263 215 reads) carried barcodes on both ends, 4.3% (213 016) contained two barcodes on the left, and 0.8% (41 128) carried two left barcodes plus a single right-end barcode. Although rare, some reads consisted almost entirely of barcodes, with up to eight in a single read (Fig. 5, available as supplementary data at *Bioinformatics* online). Overall, ≈17% of reads deviated from the expected design.

**Table 1:** Top 10 barbell patterns in rapid barcoding.^a^

	Pattern	Dorado	Flexiplex	Barbell	All
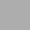	Ftag[fw, *, @left(0.0.250)]	3 971 269 (85.5%)	4 010 601 (85.9%)	4 029 358 (95.4%)	4 095 862 (83.0%)
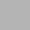	Ftag[fw, *, @left(0.0.250)]- -Ftag[fw, *, @right(0.0.250)]	258 785 (5.6%)	259 800 (5.6%)	17 251 (0.4%)	263 215 (5.3%)
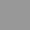	Ftag[fw, *, @left(0.0.250)]- -Ftag[fw, *, @prev_left(0.0.250)]	195 919 (4.2%)	207 737 (4.5%)	175 521 (4.2%)	213 016 (4.3%)
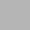	Fflank[fw, *, @left(0.0.250)]	7939 (0.2%)	28 783 (0.6%)	0 (0.0%)	94 685 (1.9%)
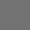	None	13 706 (0.3%)	0 (0.0%)	0 (0.0%)	91 428 (1.9%)
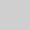	Ftag[fw, *, @left(0.0.250)]- -Fflank[fw, *, @right(0.0.250)]	51 251 (1.1%)	51 413 (1.1%)	0 (0.0%)	52 948 (1.1%)
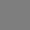	Ftag[fw, *, @left(0.0.250)]- -Ftag[fw, *, @prev_left(0.0.250)]- -Ftag[fw, *, @right(0.0.250)]	37 866 (0.8%)	40 293 (0.9%)	1901 (0.0%)	41 128 (0.8%)
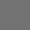	Ftag[fw, *, @left(0.0.250)]- -Fflank[fw, *, @prev_left(0.0.250)]	17 962 (0.4%)	17 378 (0.4%)	0 (0.0%)	22 141 (0.4%)
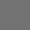	Ftag[fw, *, @left(0.0.250)]- -Ftag[fw, *, @prev_left(0.0.250)]- -Ftag[fw, *, @prev_left(0.0.250)]	8638 (0.2%)	12 785 (0.3%)	12 (0.0%)	13 178 (0.3%)
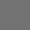	Ftag[fw, *, @left(0.0.250)]- -Ftag[fw, *, @prev_left(0.0.250)]- -Fflank[fw, *, @right(0.0.250)]	11 894 (0.3%)	12 655 (0.3%)	0 (0.0%)	13 082 (0.3%)
**Total**		4 647 221	4 667 336	4 224 099	4 937 349

a This table shows the 10 most common out of 680 total patterns detected in the reads. The first column contains the colour used in the plots throughout this manuscript. The “Pattern” column shows the read pattern assigned by Barbell (see Section 2.3). The “All” column is based on the Barbell annotate output, and the other columns show the number of reads with the pattern assigned by Barbell that were trimmed and output by Dorado, Flexiplex, and Barbell. The percentages are based on the total number of demultiplexed reads by each tool (Total). The discrepancy between the “All” count and the count in the “Barbell” column corresponds to the number of cases where the full read sequence was trimmed, e.g. when the entire read consisted of barcodes or flanks. The None pattern indicates that Barbell did not find any barcodes and thus no pattern was assigned to those reads.

**Figure 3:**
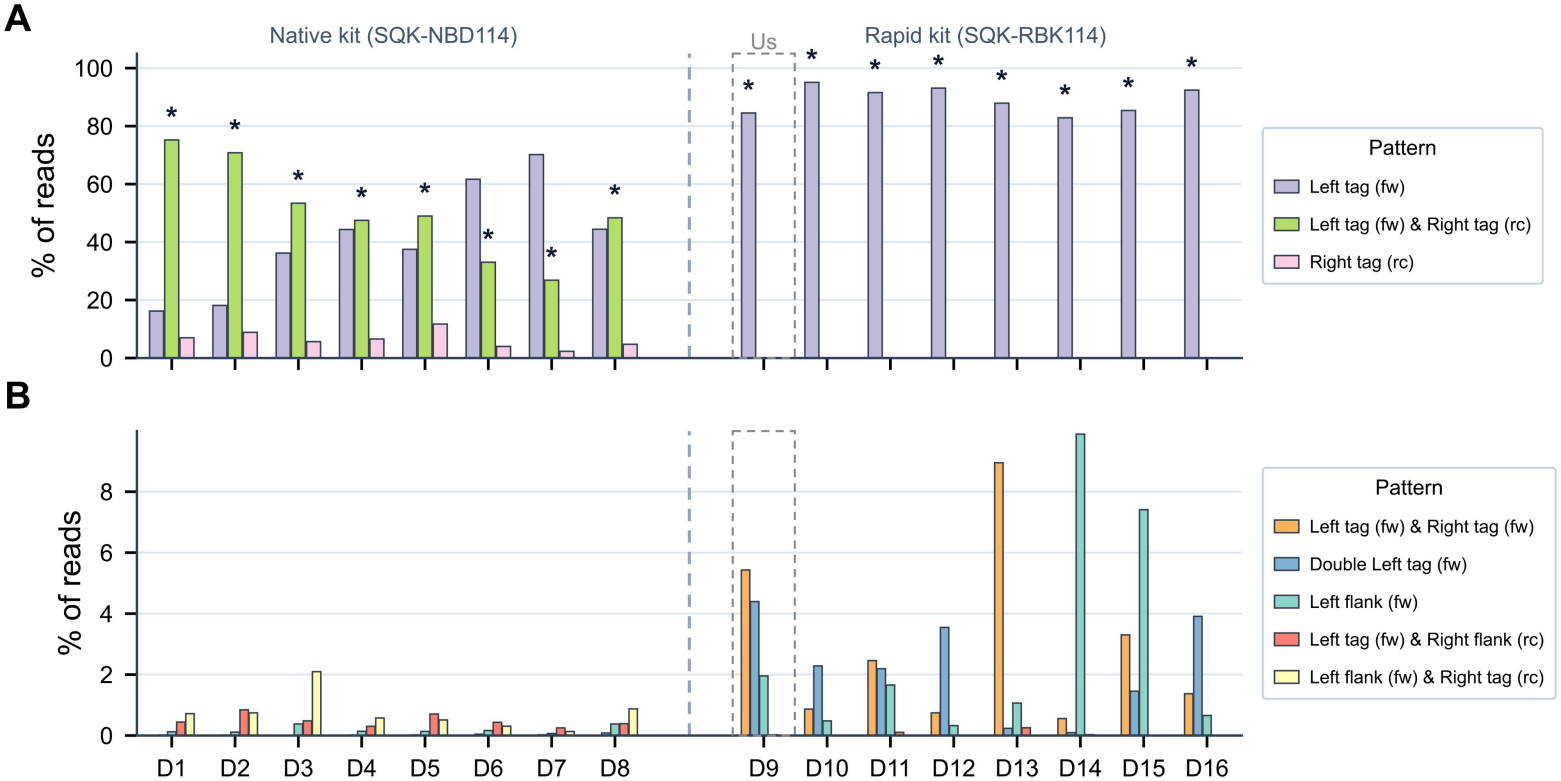
Barcode patterns in public native and rapid barcoding datasets. Barcode attachment patterns across eight rapid barcoding datasets (SQK-RBK114; including ours, dashed rectangle) and eight native barcoding datasets (SQK-NBD114) from the SRA. All datasets were analysed with Barbell using - -maximize [and -use-extended for rapid kits (Sections 2.3 and 3.2)]. Panel (A) shows the three most frequent patterns per dataset (>10% of reads), while (B) shows less frequent patterns (at most 10% of reads). The asterisk (*) above a bar indicates the expected pattern for the given kit. SRA accession numbers for each dataset (Dx) are listed in Table 2, available as supplementary data at *Bioinformatics* online.

To assess whether these deviations are consistent across rapid barcoding datasets, we used Barbell to analyse seven randomly selected rapid datasets from the SRA (Fig. 3). Across eight rapid datasets in total (including ours), the expected single barcode pattern was observed in 89% of reads on average, followed by 3% with barcodes on both ends and 2.3% with two barcodes on the left.

For context, we also analysed eight randomly selected native barcoding datasets. While not the focus of this study, these show a markedly different distribution, with the expected barcode pair on both ends observed in 50.5% of reads, followed by 41% with a barcode only on the left and 6.3% only on the right (Fig. 3). The remainder had different configurations (Fig. 3B). Note that “left” and “right” are defined by the input read orientation and may not reflect true barcode attachment bias.

#### 3.2.2 Incorrectly trimmed reads

To detect residual adapter and barcode sequences after trimming, we scanned all trimmed reads for flanks and barcodes with Sassy (Beeloo and Groot Koerkamp 2026a, b) using an edit-distance-based search (see Appendix A, available as supplementary data at *Bioinformatics* online). Nanopore adapter and barcode remnants (hereafter “contamination”) were detected in 10.0% of reads trimmed by Dorado (464 518), 8.7% by Flexiplex (406 450), and 0.004% by Barbell (166). The few contaminated reads detected after Barbell trimming arose from Sassy’s overhang-based search, which assigns lower cost to missing terminal bases and can therefore detect a second partial barcode only after trimming the first.

**Figure 4:**
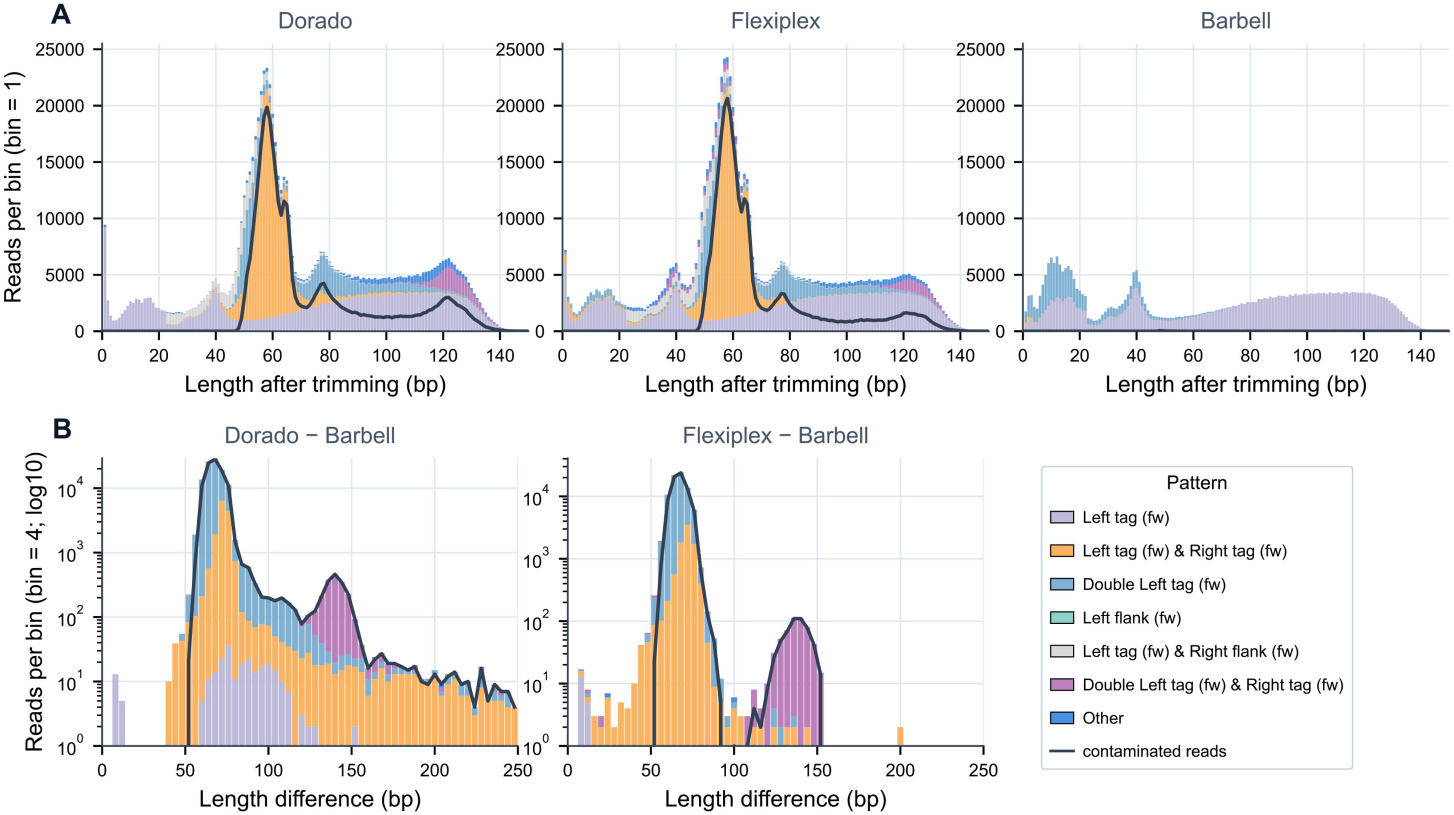
Trimmed read length comparisons. (A) Distribution of read lengths after trimming short reads (≤250 bp) for Dorado, Flexiplex, and Barbell. Bars are coloured according to the pattern assigned by Barbell, as outlined in Table 1. Most reads were trimmed from 250 bp to ≤150 bp (280 reads >150 bp not shown). Dorado and Flexiplex produced many trimmed reads of ≈60 bp, visible as a prominent peak, which were absent in Barbell output. These ≈60 bp trimmed reads originated from sequences containing two adjacent barcodes without sequence in between: Dorado and Flexiplex recognized only one barcode and output the remaining barcode sequence as a valid read, whereas Barbell detected both barcodes and removed the entire read as contamination. The black line indicates trimmed reads that contained detectable Nanopore adapter sequences (see Section 2), which closely tracked the ≈60 bp contamination peak, confirming these were artefact sequences rather than genuine biological reads. (B) Trimmed read length differences for long reads >250 bp comparing reads trimmed by Dorado versus Barbell (left) and Flexiplex versus Barbell (right). Note the logarithmic *y*-axis. Both Dorado and Flexiplex output longer trimmed reads than Barbell, often corresponding to an additional single undetected (≈60 bp peak) or two undetected (≈120 bp peak) barcodes per read, similar to those in (A). As the difference between the tools was generally ≤10 bp, we only showed differences >10 bp.

Short reads (≤250 bp; 880 637 total) were trimmed inconsistently across tools. Dorado retained 88.1% (775 409) of these reads after trimming, Flexiplex 89.0% (783 496), and Barbell 43.5% (383 320). Here, “retained” indicates reads not entirely consumed by adapter/barcode removal. Among retained short reads, contamination was found in 44.5% (345 142) of Dorado-trimmed reads, 40.3% (315 877) of Flexiplex-trimmed reads, and 0.04% (160) of Barbell-trimmed reads. In Dorado and Flexiplex, contamination was primarily associated with failure to detect additional barcodes when multiple were present (Fig. 4A). These patterns produced characteristic length peaks at ∼60 bp (one remaining barcode) and ∼120 bp (two remaining barcodes). An entire flank with barcode is 90 bp, so the remaining copy was shorter by ∼30 bp as its prefix was often partially lost in such double barcode cases (Fig. 6, available as supplementary data at *Bioinformatics* online).

Longer reads (>250 bp) were also trimmed inconsistently. Relative to Barbell, 424 207 reads trimmed by Dorado and 403 233 reads trimmed by Flexiplex were longer for the same input reads, with length differences exceeding 10 bp in 108 095 and 80 659 reads, respectively. Of these, 93.1% (Dorado) and 91.7% (Flexiplex) contained contamination (Fig. 4B). Even among reads ≥1000 bp, typically used for genome assembly, contamination was detected in 64 157 Dorado reads, 43 705 Flexiplex reads, and 3 Barbell reads. Overall, incorrect trimming affected ≈10% of reads when using Dorado or Flexiplex, mostly short reads, but persisted in long reads.

#### 3.2.3 Incorrect trimming and taxonomic assignment

Next, we evaluated how contamination affected the taxonomic assignment of reads. Since Flexiplex and Dorado showed similar trimming patterns and contamination rates (Fig. 4), we focus on Dorado comparisons for conciseness. It is the most widely adopted tool and is integrated in MinKNOW workflows. The reads trimmed by Dorado and Barbell were annotated using Centrifuger, which assigns taxonomy based on k-mer matches between reads and a reference database [e.g. RefSeq (Tatusova *et al.* 2014)]. When a read matches multiple reference entries belonging to different taxa, Centrifuger reports the lowest common ancestor (LCA) shared across all matches in the taxonomic hierarchy. As a result, reads with heterogeneous species-level matches may be assigned only at higher ranks (e.g. family level), without an associated specific RefSeq accession.

We compared RefSeq-based classifications after trimming with Dorado and Barbell. Of the 4 647 221 Dorado reads, 3 808 962 (82.0%) received a taxonomic assignment, compared to 3 588 733 of 4 224 099 Barbell reads (85.0%). At the family level, 3 370 802 Dorado reads and 3 194 077 Barbell reads were resolved. Across the 3 442 152 reads for which either tool assigned a family, 3 107 631 (90.3%) were assigned to the same family and 334 521 (9.7%) to different families. Most discrepancies arose from Barbell-trimmed reads that were unclassified at the family level, whereas the corresponding Dorado-trimmed reads were assigned to *Enterobacteriaceae* (203 122; 60.7%), including many classified as *E. coli* (48 391; 23.8%).

Among reads assigned to *Enterobacteriaceae*, 176 359 (86.8%) produced multiple RefSeq matches spanning different species, leading Centrifuger to report only the family-level LCA, as described above, without a specific RefSeq accession. The remaining 26 763 reads (13.2%) were assigned to 244 unique RefSeq entries. Analysis of the assigned reads showed that residual *Mu* transposon sequence from Nanopore Rapid Barcode flanks matched endogenous *Mu*-like elements in bacterial genomes: 24 857 (92.9%) of alignments mapped to regions characteristic of transposon- and *Mu* phage integration sites (Appendix D, available as supplementary data at *Bioinformatics* online), a result confirmed by direct alignment to the *Mu* phage genome. Thus, untrimmed tagmentation-derived *Mu* sequences created artificial matches to endogenous *Mu*-like elements, producing misleading taxonomic assignments.

The Genome Taxonomy Database (GTDB) is often used for taxonomic annotation as its high-quality sequences are expected to yield accurate assignments. Using Centrifuger with GTDB, 3 705 248 reads were annotated by either tool, of which 346 016 (9.3%) were assigned to a different family. Where Barbell’s reads were unassigned, Dorado’s reads were mostly assigned to *Balneolaceae* (103 547; 29.9%) and *Streptomycetaceae* (98 098; 28.3%). These assignments originated from read contamination matching contamination in public assemblies (see Section 3.4). Specifically, 67.4% of *Streptomycetaceae*, all *Streptomyces* species, were assembled by Jørgensen *et al.* (2024). All *Balneolaceae* were from a single *Gracilimonas* assembly (GCF_040117685.1) by Lim *et al.* (2025). Notably, the matched regions included both *Mu* transposon flanks and Nanopore barcode sequences, making an endogenous *Mu*-like origin implausible. Because the rapid barcoding region spans only 90 bp (see Section 2), restricting taxonomic assignments to matches ≥100 bp reduced rapid barcoding contamination, lowering family-level discrepancies to 129 reads at the cost of a ≈25% reduction in assigned reads.

#### 3.2.4 Double barcode attachment and bleeding

Double left-end barcodes were detected in 213 016 reads (4.3% of total; Table 1). In 100 042 (47.0%) of these, the right flank of the first barcode was directly fused to the second barcode, resulting in loss of the left flank and partial deletion of the second barcode (Fig. 6, available as supplementary data at *Bioinformatics* online). Consequently, the mean edit distance to the second barcode was higher than to the first (7 versus 3). Fusion-associated deletions varied by barcode and were also observed in public datasets (Appendix E, available as supplementary data at *Bioinformatics* online). Barbell users can enable fusion detection using the - -use-extended option.

Of the reads with double barcodes, 212 048 (99.5%) carried the same barcode twice and therefore did not affect demultiplexing, although being the main source of trimming errors. In the remaining 968 reads (0.5%), the tools disagreed: Dorado selected the outer barcode copy, whereas Barbell selected the inner copy under the assumption that the first tagmentation was the correct one. Read-level taxonomic annotation supported the Barbell assignment in 53.2% of cases, compared to 4.4% support for the Dorado (outer-barcode) assignment.

#### 3.2.5 Propagation into assemblies

Assemblies were successfully generated for 64 of 66 samples; the remaining two contained too few reads for assembly. Across these 64 assemblies (59 bacterial and 5 fungal; see Table 1, available as supplementary data at *Bioinformatics* online), no systematic differences in continuity were detected between assemblies from Barbell and Dorado trimmed reads (see Section C.1, available as supplementary data at *Bioinformatics* online; paired Wilcoxon signed-rank test, *P* = .22). For the bacterial subset, CheckM2 reported near-identical quality metrics: Dorado assemblies had mean completeness of 99.34% and mean contamination of 1.00%, whereas Barbell assemblies had mean completeness of 99.35% and mean contamination of 1.00%.

However, CheckM2 estimates contamination using single-copy marker genes and can therefore fail to detect non-biological, experiment-specific artefacts originating from library preparation. To identify these artefacts, we screened all 64 assemblies for residual rapid-barcoding sequence. Contamination was detected in seven assemblies from Dorado-trimmed reads, and in none of the assemblies produced from Barbell-trimmed reads. For example, a contig from a *Saccharomyces cerevisiae* assembly contained a 92-bp segment that did not occur in canonical *S. cerevisiae* genomes but instead matched diverse bacterial and synthetic sequences—a leftover produced when Dorado trimmed only the first of two barcode occurrences (Fig. 7, available as supplementary data at *Bioinformatics* online). Thus, although marker-based completeness/contamination metrics were similar, Barbell effectively removed experimental artefacts that can confound taxonomic annotation.

### 3.3 Comparing scoring schemes

Overall, Dorado demultiplexed 456 953 more reads than Barbell. Of these, 92% (428 823) were reads that Barbell had detected but excluded from its final output because they failed to match expected rapid-barcoding patterns or were trimmed to empty sequences.

To assess the accuracy of these additional assignments, we compared species-level taxonomic annotations of trimmed reads with those of the assemblies associated with their assigned barcodes. Only 30 308 (6.6%) of the additional Dorado-demultiplexed reads were taxonomically consistent, indicating that most were incorrectly assigned. Of those consistent with taxonomy, 21 645 were excluded by Barbell due to conservative scoring. This included 13 706 reads whose barcoding flanks exceeded the default edit-distance cutoff and 7939 reads annotated as Fflank due to inconclusive subsequence scoring.

Conversely, Barbell demultiplexed 86 609 reads that Dorado failed to assign. Of these, 60 009 (69.3%) were taxonomically consistent with their corresponding assemblies, with the remaining reads being taxonomically unclassified.

Note that the comparison in this section focuses on the quantitative aspect of demultiplexing (i.e. the number of assigned reads), whereas the earlier trimming analysis (Section 3.2) evaluated qualitative accuracy (i.e. removal of contamination). Overall, Barbell recovered 60 009 taxonomically consistent reads that Dorado failed to assign while missing 30 308 reads that Dorado likely demultiplexed correctly. However, as shown previously, 10% of Dorado-demultiplexed reads retained barcode contamination, compared to 0.004% for Barbell, demonstrating that high assignment rates do not necessarily reflect higher quality.

### 3.4 Barcodes and their flanks in public databases

Adapter contamination in genome assemblies has previously been reported for Illumina data (Moeller *et al.* 2024). Because we observed that Nanopore sequences are not always removed by standard tools such as Dorado, we queried the NCBI “core nucleotide” database (≈810 GB) for Nanopore barcode and flank sequences.

#### 3.4.1 Overview

We found detectable footprints of both rapid and native Nanopore barcoding kits across viruses, bacteriophages, bacteria, plasmids, organelles, and rRNA records. Filtered match tables have been uploaded to Zenodo (Filtered match results can be found at https://zenodo.org/records/17396505). Read-level re-analyses (read counts, trimming parameters, and assembly commands) are provided in Appendix G, available as supplementary data at *Bioinformatics* online and summarized below.

#### 3.4.2 Rapid barcodes

For rapid kits we observed 103 matches where both rapid barcode flanks and barcode sequences were present, including 68 exact (0-edit) matches across 67 assemblies; additional hits to flanks alone were excluded because they may reflect endogenous *Mu* transposons (Section 3.2). The most striking example was *Photobacterium leiognathi* SV5.1 (CP131573.1), where BC86 occurred 11 times across a 1.43 Mb contig. The barcodes were scattered internally, consistent with scaffolding of smaller contigs separated by N stretches. Repeating the demultiplexing and trimming of the raw reads using Barbell (see Appendix G, available as supplementary data at *Bioinformatics* online) followed by reassembly removed barcode sequences and produced contigs consistent with the expected chromosomal arrangement. Another clear case was an *E. coli* plasmid (CP165501.1) carrying BC10 and BC09 overhangs at opposite ends of the contig. The sample was annotated as a BC10 sample, but Barbell revealed barcode bleeding with 13.1% of the raw reads from the BC09 sample. Re-assembly from Barbell reads for just BC10 eliminated the barcode contamination. Other rapid-kit examples included plasmids, mobile elements, and assemblies from Jørgensen *et al.* (2024), *Streptococcus thermophilus* (CP072431.1), and *Staphylococcus aureus* (CP150769).

#### 3.4.3 Native barcodes

For native kits we detected 462 matches to native flanks and barcodes, of which 270 were exact matches across 284 assemblies. Native-kit contamination was widespread and spanned viruses (including human SARS-CoV-2; OV192362.1), bacteriophages (OP583592, OR487170.1, PP989835.1), bacteria (e.g. *Mycolicibacterium novocastrense*, CP097264.1—50 matches to NB02), parasites, fungi, short rRNA records, and organellar genomes (mitochondrial *Tonna galea*, NC_082277; chloroplast *Cephaleuros karstenii*, NC_060534). For example, an 8423 bp plasmid (CP142556.1) contained a remnant NB13 barcode at the contig end. Despite being annotated as circular, self-alignment showed an overlap leaving the barcode as an overhang, and subsequent Illumina polishing by the authors did not remove the artefact (Li *et al.* 2024), demonstrating that circularity calls alone do not guarantee contamination-free sequences.

Thus, both rapid and native Nanopore barcoding kits have left detectable, and sometimes assembly-affecting, footprints in public sequence databases. Careful read-level trimming and reassembly removed barcode-derived artefacts.

## 4 Discussion

Barbell is a powerful demultiplexing tool that provides insight into the adapter and barcode patterns in Nanopore reads, many of which may result from experimental artefacts. It substantially reduced trimming errors and minimized barcode bleeding.

### 4.1 Barcode patterns

A large portion of research on Nanopore sequencing focuses on establishing error rates and mitigating their effects in downstream analysis by generating consensus sequences (Greenfield *et al.* 2014, Rang *et al.* 2018, Liu *et al.* 2024). However, much less attention has been paid to what happens to reads during the experimental steps such as tagmentation and ligation. Only ≈80% of reads in our rapid barcoding experiments were of the expected configuration, with other public experiments showing similar patterns (average 89%). The remaining ≈20% contained multiple barcodes in different configurations.

Specifically, reads having two barcodes on the left or barcodes on both ends of the read are problematic for existing tools (Fig. 4). For double-left barcodes, the second copy often lacked the entire left flank and a partial prefix of the second barcode (Fig. 3, available as supplementary data at *Bioinformatics* online). As for the physicochemical mechanism underlying these sequence anomalies, we did not observe any abnormal spikes in the pore signal that would indicate secondary structure (Appendix F, available as supplementary data at *Bioinformatics* online). An alternative hypothesis is that there are biases in Nanopore sequencing related to the *Mu* target site (Chen *et al.* 2025). However, we did not conclusively identify a signature explaining this phenomenon, and further investigation is warranted. Such fusions are difficult to demultiplex for two reasons. First, the entire loss of the left flank makes it difficult to locate the barcode region in the first place, and second, prefix loss of the barcode increases its edit distance to the correct barcode and potentially lowers it to other barcodes. To address this, we added the fusion pattern to Barbell (enabled by --use-extended).

While identical double barcodes on the left side heavily impaired trimming by Dorado, around 0.5% of the double left reads had two different barcodes. Dorado’s selection of the outer barcode here is likely a source of barcode bleeding based on the taxonomy analysis.

### 4.2 Contamination

Contamination from reagents and kits is well known to affect downstream analyses (Salter *et al.* 2014). Many metagenomes in the MGnify database contain Illumina adapter contamination (Moeller *et al.* 2024), and we demonstrated that Nanopore barcodes and their flanking sequences can also appear in assemblies when using existing demultiplexers. Moreover, our analysis of the “core nucleotide” database suggests that many public assemblies contain Nanopore contamination. A valuable next analysis would be to repeat such analyses for other public resources such as GTDB, which is commonly used for taxonomic assignment. The presence of such contamination can generate spurious taxonomic signals, particularly when untrimmed barcodes and flanks from demultiplexers form misleading links with contamination in the public databases (Section 3.2). Therefore, researchers should exercise caution when using public data as reference material. Special attention should be paid to whether matches stem from barcodes or flanking regions. Stricter post-processing rules—e.g. requiring matches of at least 100 bp—can help reduce such spurious matches.

Related to the public database analysis (Appendix D, available as supplementary data at *Bioinformatics* online), we note that the rapid kit (SQK-RBK114-96) uses *Mu* transposon sequences to tagment barcodes. Since these *Mu* transposons naturally occur in several *Enterobacteriaceae* species, they can yield apparent “false positive” Fflank matches; conversely, residual flanking sequence may be annotated as a *Mu* transposon. Although such reads can still be included by allowing Fflank matches within reads, it is important to be aware of these potential effects.

Researchers are increasingly developing custom experiments using their own barcodes, primers, or other tags, often resorting to custom demultiplexing scripts. Barbell is specifically designed for such cases as a modular tool, where the flank and barcode sequences can be changed to fit the user’s needs without requiring users to write their own scripts or code. This functionality supports experimental design, helps detect potential issues, and enables subsetting of reads based on expected patterns.

## 5 Conclusion

We demonstrated that commonly used demultiplexers leave ∼10% of Nanopore reads improperly trimmed in our dataset, which can significantly impact downstream analyses such as taxonomic annotation and genome assembly. These effects are further exacerbated by the presence of similar contamination in public databases, including the core nucleotide database and GTDB, which can create artificial connections lacking true biological meaning. Barbell detected complex barcode patterns and reduced barcode bleeding and trimming errors.

## Supplementary Material

btag349_Supplementary_Data

## Data Availability

Generated reads are available under BioProject PRJEB100828. All code, reproducible analysis steps, and public data search results are available on Zenodo (https://doi.org/10.5281/zenodo.19494282, https://doi.org/10.5281/zenodo.17396505). The Barbell version used to generate the results is also available on Zenodo (https://doi.org/10.5281/zenodo.19493956).
