# Supplementary material for: Barbell reveals and resolves demultiplexing and trimming issues in Nanopore data
Source: Bioinformatics. 2026 Jun 2;42(6):btag349. doi: 10.1093/bioinformatics/btag349 (PMC13293122; doi:10.1093/bioinformatics/btag349)
Supplement: btag349_Supplementary_Data [file btag349_supplementary_data.pdf]

A. Further methods

A.1. Rapid barcoding patterns

For rapid barcoding we will consider the following two patterns safe:

1. `Ftag[fw,*,@left(0..250),>>]`
2. `Ftag[fw,?1,@left(0..250)]__Ftag[fw,?1,@prev_left(0..250),>>]`

The first pattern is the ideal pattern, with just a single left tag. The second pattern covers the second most common pattern (later in Results), in which two barcodes are ligated, and we take the label from the inner tag (the one with `>>`), although we do enforce that both barcodes are the same using the `?1` wildcard.

In case we want to maximise (`--maximize`) matches potentially at the expense of accuracy, we add the following patterns:

1. `Ftag[fw,*,@left(0..250)]__Ftag[fw,*,@prev_left(0..250),>>]`
2. `Ftag[fw,*,@left(0..250),>>]__Ftag[<<,fw,*,@right(0..250)]`
3. `Ftag[fw,*,@left(0..250)]__Ftag[fw,*,@prev_left(0..250),>>]__Ftag[<<,fw,*,@right(0..250)]`

Here we always determine the sample based on the inner barcode, but are more flexible by allowing additional barcodes to be present. Using maximise patterns will give the highest yield and should be used for tasks such as assembly. However, for diagnostics and quantification, where false positives may affect the outcome, it might be better to use just the safe patterns.

A.2. DNA isolation and sequencing

66 unidentified bacterial and fungal isolates were selected for Nanopore sequencing for diagnostic purposes (Table S1). Briefly, genomic DNA was isolated using the DNeasy Ultra Clean Microbial kit (Qiagen, Venlo, the Netherlands). Nanopore sequencing was performed according to the rapid barcoding protocol RBK96.114 on an R10.4.1 flow cell with MinION (Oxford Nanopore, Oxford, UK). Bases were called using super accurate basecalling using MinKNOW v24.11.10.

A.3. Sequence searching and assemblies

For the analyses, we used Sassy [Beeloo and Groot Koerkamp, 2026a] to search based on edit distance, always using an overhang of $\alpha = 0.5$ (`-a 0.5`) to find matches crossing read boundaries and the IUPAC alphabet to handle ambiguous bases (`--alphabet iupac`). To identify rapid barcoding contamination, we require that—aside from the flanks—a barcode is detected within ≤ 4 edits. Rapid barcoding kits use the *Mu* transposase for barcode and adapter attachment. Since the *Mu* transposase is naturally encoded by the *Mu* phage, which infects *Enterobacteriaceae*, searching for just the flank could produce false positive matches—cases where the match reflects the presence of *Mu* phage rather than true contamination. Annotation of our genomes showed two *Enterobacteriaceae* species in our own dataset. Moreover, when we later searched databases, these included many *Enterobacteriaceae* species.

As noted previously (Section 2.1), a typical rapid barcode flank consists of a left flank (F_ℓ), a barcode (B), and a right flank (F_r). In experimental data, however, we observed reads with two barcodes on the left side, following this concatenation configuration:

$$F_\ell \circ B \circ F_r \circ B \circ F_r.$$

In this case, the second barcode entirely lacks its left flank and instead appears directly adjacent to the right flank of the preceding barcode region. We refer to such structures as fusions, and we searched for them in our datasets using the pattern GTTTTTCGTGCG CCGCTTCA<barcode_seq>GTTTTCGCATTTATCGTGAAACG. To detect fusions, we used MMseqs2 [Steinegger and Söding, 2017] using the following parameters: `-search-type 3`, `-max-seqs 5000000`, `-max-seq-len 200000`. We initially used MMseqs2 instead of Sassy, since it was unclear whether fusion events would appear primarily as semi-global matches or also as shorter local sub-matches.

Typically, Filtlong (`https://github.com/rrwick/filtlong`) is used to discard the worst 10% of reads prior to assembly. Since this depends on how well the demultiplexer has already removed low-quality reads, we instead applied absolute thresholds. Reads were filtered with Filtlong (`v0.2.1`) keeping those $\geq$ 1000 bp (`-min_length 1000`) and with mean quality $\geq$ 15 (`-min_mean_q 15`). To assemble the genomes we used Flye (`v2.9.6-b1802`) [Kolmogorov et al., 2019] in `-ont-hq` mode with 5 polishing iterations (`-i 5`), followed by a final polishing using Medaka (`https://github.com/nanoporetech/medaka`). To map sequences to assemblies we used minimap2 (`v2.28-r1209`) [Li, 2018], in `map-ont` mode (default parameters). To compare assemblies, we first extracted the contigs using Samtools [Li et al., 2009], then mapped these to each other using Minimap2 (`map-ont`) [Li, 2018], followed by graph induction using seqwish [Garrison and Guarracino, 2022] and visualised using Bandage [Wick et al., 2015].

A.4. Taxonomic annotation

We used Centrifuger [Song and Langmead, 2024] with the RefSeq database [Pruitt et al., 2007] and Genome Taxonomy Database (GTDB) (`r226`) [Parks et al., 2021]. As the GTDB alone does not include fungal sequences, we used the pre-built GTDB + fungi database provided by Centrifuger. To link taxonomy identifiers to taxonomic lineages we used ete3 [Huerta-Cepas et al., 2016].

B. Subsequence barcode scoring scheme

Motivation. In Nanopore sequencing, errors often occur as localised stretches of incorrect or missing nucleotides, typically caused by slippage or transient stalling of DNA in the pore [Delahaye and Nicolas, 2021]. A single error stretch (e.g. TTTT) can already introduce multiple consecutive edits in an otherwise correct alignment. In contrast, observing the same number of edits scattered across an entire barcode is much less likely to result from such localised effects. Consequently, a scoring scheme that rewards compact groups of matches provides improved discrimination between true barcode matches and alignments dominated by localised error runs (see Figure S1).

Setup. Given an edit-distance alignment between a barcode and a read, we consider its CIGAR string C. From C we extract the query positions that are exact matches (i.e. not substitutions, insertions, or deletions), and index them in increasing order as

$$P = (p_1 < p_2 < \cdots < p_{|P|}).$$

Let $k \geq 1$ be a subsequence length and let $\lambda \in (0, 1]$ be a decay parameter.

Score definition. The subsequence score $S_k(C;\lambda)$ counts all increasing k-tuples of match positions and weights each tuple by an exponential penalty that depends on its span. Formally,

$$S_k(C;\lambda) \;=\; \sum_{1\le i_1<i_2<\cdots<i_k\le |P|} \lambda^{\,p_{i_k}-p_{i_1}+1}.$$

If $|P|<k$, we define $S_k=0$. Smaller values of λ penalise widely spaced matches more strongly, whereas values of λ close to 1 treat spacing more uniformly. Intuitively, compact runs of k matches contribute substantially more to the score than the same number of matches distributed across a larger region of the barcode.

Efficient evaluation. Following the subsequence kernel formulation of Lodhi et al. [2002], we compute S_k using dynamic programming on the match positions. Let $D_i^{(t)}$ denote the total weight of all increasing t-tuples whose last element is p_i. Then

$$D_i^{(1)} = \lambda,$$

$$D_i^{(t)} = \sum_{j<i} D_j^{(t-1)}\,\lambda^{\,p_i-p_j}, \qquad t=2,\dots,k.$$

The final score is obtained as $S_k=\sum_i D_i^{(k)}$. In practice, $|P|$ is bounded by the barcode length, making this computation feasible for typical parameter choices. While this approach could arguably be used to directly search both flanks and barcodes, it is prohibitively slow compared to edit distance, which can be accelerated using SIMD instructions; see Beeloo and Groot Koerkamp [2026a,b] for more details. An implementation operating directly on CIGAR strings is available in our crate.[5]

Usage. In this work, edit distance is used to locate τ_N within a read, while subsequence scoring is applied in a second stage to discriminate between candidate barcodes. This separation allows us to retain computational efficiency during localisation while exploiting the increased sensitivity of subsequence scoring during barcode assignment.

Full calculation of CIGAR examples.

We illustrate the score for $k=3$ with decay λ on two CIGAR strings. Given match positions $\textsc{pos}=(p_1<\cdots<p_{|C|})$, the score is

$$K_3(C;\lambda) \;=\; \sum_{1\le i<j<\ell\le |C|} \lambda^{\,p_\ell-p_i+1}.$$

There is exactly one triple when $|C|=3$, namely $(i,j,\ell)=(1,2,3)$, so $K_3=\lambda^{\,p_3-p_1+1}$.

Example 1: `Sub Sub Match Match Match.` (Figure S1A) Advancing the alignment index by each operation yields match positions $\textsc{pos}=(2,3,4)$. The only 3-subsequence is $(2,3,4)$ with inclusive span $4-2+1=3$, hence

$$K_3 \;=\; \lambda^3.$$

For $\lambda=\frac{1}{2}$, $K_3=2^{-3}=\frac{1}{8}=0.125$.

Example 2: `Match Sub Match Sub Match.` (Figure S1B) Match positions are $\textsc{pos}=(0,2,4)$. The only 3-subsequence is $(0,2,4)$ with inclusive span $4-0+1=5$, hence

$$K_3 \;=\; \lambda^5.$$

For $\lambda=\frac{1}{2}$, $K_3=2^{-5}=\frac{1}{32}=0.03125$.

[5] `https://github.com/rickbeeloo/cigar-lodhi-rs.git`

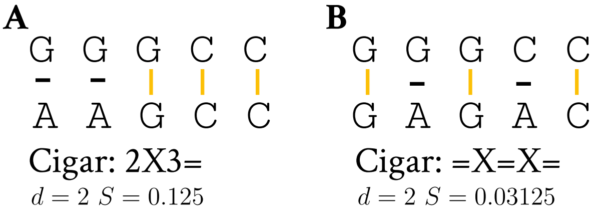

Fig. S1: Example of edit distance and subsequence scoring. Both alignments here have the same edit distance of 2, but in **(A)** the matches and errors are continuous, whereas in **(B)** the matches and errors are interleaved. Considering Nanopore errors arising from slippage and stalling of DNA in the pore, the alignment in **(A)** is more likely to be correct, which is captured by the subsequence scoring (S).

Interpretation. Both examples contain exactly one ordered triple of matches; the difference is the spacing between the first and last matches. The second CIGAR has larger gaps (due to substitutions), increasing the span and thus down-weighting the contribution more strongly via the λ^{span} factor.

Parameter choice. Throughout this work we use $k=3$ and $\lambda=0.5$. These values were selected heuristically to capture short, locally consistent runs of matches, which are characteristic of correct Nanopore alignments in the presence of localised error bursts (see Examples 1 and 2 above, where these parameters yield a $4\times$ score difference between compact and dispersed alignments of identical edit distance). Since the score is used only for relative ranking of candidate barcodes, the exact values of k and λ are not critical: for any $\lambda\in(0,1)$ and $k\ge 2$, a compact alignment will always score higher than a dispersed one with the same number of matches, preserving the ranking regardless of the specific parameter choice. We therefore did not perform fine-grained parameter tuning.

C. Cut-offs for flank and barcode matching

A key step in demultiplexing is determining thresholds that decide whether a region matching the flank τ_N and barcode B should be accepted as a true match. As in other tools, Barbell uses edit distance to locate τ_N, but employs subsequence scoring for barcode discrimination. Because both measures depend on sequence length and scoring parameters, cut-offs are derived automatically and reported to the user.

Edit-distance cut-off for flank localisation.. The expected edit distance between two random strings is approximately 51% of their length, with observed values typically ranging between 36% and 63% [Rosenfeld, 2024]. In rapid barcoding, the flank τ_N has length $|\tau_N|=90$, but contains an internal mask $N_{|B|}$ of 24 N characters that match any base. We therefore define the effective flank length as

$$|\tau_N|_{\text{eff}} = |\tau_N| - |B| = 66.$$

Using the theoretical lower bound, one would expect approximately $66\cdot 0.36\approx 24$ edits when aligning a random string to the effective flank. However, these bounds are derived from simulations on long sequences and are overly permissive for shorter tags. To address this, we empirically fitted a lower bound to edit-distance distributions obtained from random short strings (see

Figure S3), yielding

$$\theta_{\text{emp}}(a) = \max\left(0,\ \left\lceil 0.51 \cdot a - 1.7312 \cdot \sqrt{a} \right\rceil\right).$$

Here, $\theta_{\text{emp}}(a)$ denotes the maximum allowed edit distance for a sequence of length a, and the subscript "emp" indicates that this bound is empirically derived. The $\sqrt{a}$ term adjusts the theoretical 51% error rate downward for shorter sequences, imposing a stricter cut-off that gradually relaxes as sequence length increases.

Applying this formula to the effective flank length gives

$$\theta_{\text{emp}}(66) = 20.$$

We verified that using the theoretical value of 24 edits results in false positive flank matches within reads, whereas the empirically derived cut-off of 20 avoids these cases. Barbell therefore uses $\theta_{\text{emp}}(a)$ to set the flank edit-distance cut-off automatically based on the length of the user-provided tags. The selected cut-off is reported to the user and can be manually adjusted if more or less stringent matching is desired (Section 2.5).

Cut-offs for barcode assignment using subsequence scores. Existing tools such as Dorado and Flexiplex use edit-distance-based heuristics to assign barcodes after locating the flanks. Flexiplex enforces a maximum edit distance (6 in this manuscript) and discards reads when multiple barcodes share the same cost. Dorado applies a more complex heuristic that restricts barcode searches to expected locations and requires the top-scoring barcode to be sufficiently separated from the second-best hit.[6]

In contrast, Barbell scores barcode regions using the subsequence scoring scheme described in Appendix B. Because the subsequence score $S_k(C;\lambda)$ depends on the parameters k and λ, its absolute value is not directly comparable across settings. To make score thresholds interpretable, we normalise scores relative to the maximum achievable score for a perfect alignment. Specifically, we compute the perfect score corresponding to a CIGAR string consisting entirely of matches. For barcodes of length 24, this corresponds to a CIGAR string of 24=. Barcode matches are then accepted if their score exceeds a user-defined fraction $S_{\min}$ of this perfect score, and if the difference between the best and second-best barcode scores exceeds an absolute fraction S_{diff}. By default, we use $S_{\min} = 0.2$ and $S_{\text{diff}} = 0.1$. To give an intuition for how these values affect the number of demultiplexed reads, and the percentage of correct reads, we show a grid in Figure S2. This normalisation allows users to reason about barcode confidence in intuitive percentage terms, while retaining the error sensitivity of subsequence scoring.

C.1. Comparison of assembly contiguity between Dorado and Barbell

Assembly contiguity for Barbell- and Dorado-trimmed reads was assessed using QUAST-style contiguity profiles. In general, assembly contiguity can be summarised using NGx curves (and corresponding area-under-the-curve metrics such as auNG), which report contig length as a function of genome coverage percentile and require knowledge of the expected or reference genome size. Because an expected genome size was not available for these samples, true NGx curves could not be computed.

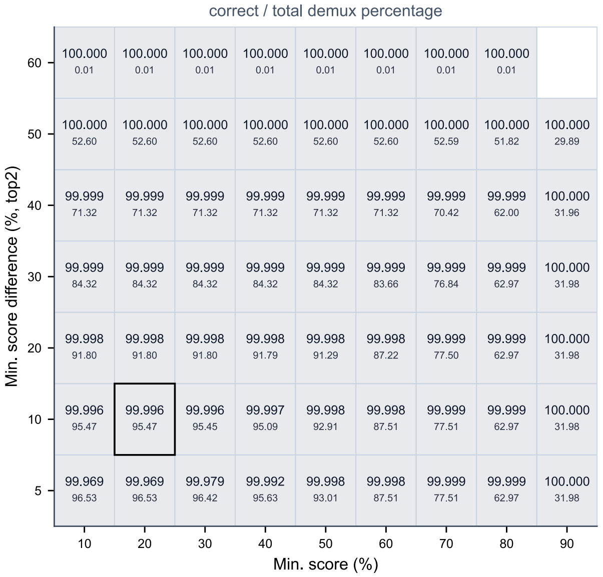

Fig. S2: $S_{\min}$ **score versus** S_{diff} **grid**. We first select 788,672 reads from our data where we had long hits to taxonomy entries using Centrifuger (min. trimmed length 250 bp) or barcode matches with edits unlikely due to chance (max 4 edits). Note that *trimmed* here is not based on any tool to avoid biases, instead we removed the first 200 bp of each read to certainly remove rapid adapters/barcodes ($\approx$ 90 bp). Then we ran Barbell on these reads varying the minimum score ($S_{\min}$; x-axis) and the minimum score difference between the top 2 matches (S_{diff}; y-axis). The former being the minimum subsequence score to assign the barcode, and the latter the difference between the top two best scoring barcodes (Algorithm 1). The upper value in each cell is the percentage "correct" (i.e. correct barcode based on taxonomy or low edit distance), and the bottom is the percentage of the total reads demultiplexed. The default cut-off is shown in the bold-bordered cell. Note that the remainder, total - correct, does not necessarily indicate an "incorrect" demultiplexing, as some reads receive unexpected barcodes (e.g. after pooling), could not be taxonomically annotated, or had high edit distances. Nevertheless, this grid provides a useful overview of how much change can be expected when varying either cut-off.

Instead, we constructed cumulative contig length–rank curves by ordering contigs from longest to shortest and plotting cumulative assembled sequence length as a function of contig rank. Each curve was normalised by the total assembly length for that barcode so that the y-axis represents the fraction of the assembly accumulated. To focus on biologically meaningful contiguity and to reduce sensitivity to very small contigs, we summarised each curve by the area under the curve up to 95% of the assembled sequence ($\text{AUC}_{0.95}$).

Because the x-axis is contig rank, $\text{AUC}_{0.95}$ has units of contigs and can be interpreted as the average number of contigs required to recover the first 95% of the assembly. Differences in $\text{AUC}_{0.95}$ therefore reflect shifts in how rapidly assembled sequence accumulates into long contigs, conditional on the assembled sequence, rather than differences in total assembly size or completeness. Higher $\text{AUC}_{0.95}$ values indicate assemblies in which more contigs are required to reach the same fraction of the assembly, consistent with greater fragmentation.

[6] As there is no dedicated publication for Dorado, see the source files `barcode_kits.h` and `BarcodeClassifier.cpp` in the Dorado GitHub repository.

Barcode	Species	Barcode	Species
barcode01	*W11650 sp030535295*	barcode34	*Cutibacterium acnes*
barcode04	*Malassezia restricta*	barcode35	*Fusobacterium russii*
barcode05	*Castellaniella denitrificans*	barcode36	*Mycobacterium abscessus*
barcode06	*Psychrobacter sanguinis*	barcode37	*Yamadazyma tenuis*
barcode07	*JAUMYT01 sp030528525*	barcode38	*Mycobacterium smegmatis*
barcode08	*QD2021 sp036209505*	barcode40	*Clostridium sp036643715*
barcode09	*Exiguobacterium_A sp038006045*	barcode41	*QD2021 sp036209505*
barcode10	*Muribacter muris*	barcode42	*Actinobacillus_C sp020026155*
barcode11	*Acinetobacter terrestris*	barcode43	*Mannheimia granulomatis*
barcode12	*Pasteurella felis*	barcode44	*Yersinia pestis*
barcode13	*Prescottella sp032085135*	barcode45	*Chelonobacter testudinis*
barcode14	*Psychrobacter sanguinis*	barcode46	*Brucella melitensis*
barcode15	*Acinetobacter sp947627655*	barcode47	*Actinobacillus_C sp020026155*
barcode16	*Capnocytophaga catalasegens*	barcode48	*Micrococcus luteus*
barcode18	*Frederiksenia canicola*	barcode49	*Saccharomyces cerevisiae*
barcode19	*Granulicatella balaenopterae*	barcode50	*Rodentibacter trehalosifermentans*
barcode20	*Granulicatella balaenopterae*	barcode51	*Actinomyces denticolens*
barcode22	*Planococcus glaciei*	barcode52	*Brevibacterium gallinarum*
barcode23	*Fastidiosipila sp963510375*	barcode53	*Streptococcus equi*
barcode24	*Brachybacterium conglomeratum*	barcode54	*Mannheimia haemolytica*
barcode25	*Prescottella equi*	barcode56	*Buchananella hordeovulneris*
barcode26	*Prescottella equi*	barcode57	*Staphylococcus simulans_B*
barcode27	*Mannheimia haemolytica*	barcode59	*QD2021 sp036209505*
barcode28	*Carnobacterium maltaromaticum*	barcode60	*Bisgaardia hudsonensis*
barcode29	*Nicoletella semolina*	barcode61	*Burkholderia thailandensis*
barcode30	*Capnocytophaga stomatis*	barcode62	*Intestinirhabdus alba*
barcode31	*Gordonia sp016919385*	barcode63	*Actinomyces denticolens*
barcode32	*Berryella intestinalis*	barcode64	*Streptococcus pasteurianus*
barcode33	*Berryella intestinalis*	barcode65	*Streptococcus gallolyticus*
		barcode66	*Streptococcus pasteurianus*

Table S1. List of barcodes and their corresponding species assignments based on Centrifuger (GTDB+Fungi), sorted by barcode. barcode02 and barcode03 are not included in the table due to insufficient reads for assembly, which prevented taxonomic annotation. We note that barcodes barcode44 and barcode46 are likely incorrectly annotated by Centrifuger and should be *Yersinia pseudotuberculosis* and *Brucella ceti*, respectively, based on more extensive analysis using an in-house pipeline. Names like *JAUMYT01* and *QD2021* are GTDB placeholders and full taxonomic information is available at GTDB e.g. `https://gtdb.ecogenomic.org/searches?s=al&q=QD2021`.

For each barcode, differences in contiguity between Dorado- and Barbell-based assemblies were calculated as $\Delta\mathrm{AUC}_{0.95} = \mathrm{AUC}_{0.95,\mathrm{Dorado}} - \mathrm{AUC}_{0.95,\mathrm{Barbell}}$. Across the 64 shared barcodes, $\Delta\mathrm{AUC}_{0.95}$ values were centred at zero (median $\Delta\mathrm{AUC}_{0.95} = 0$; mean -0.078; bootstrap 95% confidence interval for the median: $[0, 0]$), indicating no systematic difference in contiguity between tools (Supplementary Fig. S4). Although individual barcodes showed larger positive or negative differences, these deviations were balanced in both directions and did not result in a significant overall shift in contiguity (paired Wilcoxon signed-rank test; $p = 0.22$).

D. Mu phage integration

To investigate the source of ambiguous *Enterobacteriaceae* assignments among trimmed Dorado reads, we examined whether residual *Mu*-transposon sequences from the Nanopore Rapid Barcode flanks might have matched endogenous *Mu* transposons in these bacteria. The 26,763 Dorado-trimmed reads were aligned to the 244 RefSeq genomes, and the genomic regions within 5 kb of the alignment sites were analysed. Most alignments (24,857 reads; 92.9%; see Section 3.2) were located near genes characteristic of transposons and nearby cargo, including *tail fibre assembly protein* (24,154 reads), *recombinase family protein* (24,153), *Mom family adenine methylcarbamoylation protein* (24,152), and *tail fibre protein* (24,152).

E. Double barcode attachment and bleeding

As reported in Section 3.2, 213,016 reads (4.3%) carried two left-end barcodes, of which 100,042 (47.0%) showed a direct barcode fusion with higher mean edit distance to the second barcode than to the first (7 vs. 3; Figure S6). Fusions were observed across all barcodes, but the prevalence of fusion-associated deletions in the first 6 bp of the second barcode varied by barcode, for example: BC05, 95.4% (2,073/2,173); BC25, 95.8% (2,106/2,198); BC61, 29.5% (901/3,053); and BC45, 52.3% (2,027/3,874). We observed similar patterns when analysing public datasets (Weinmaier et al.

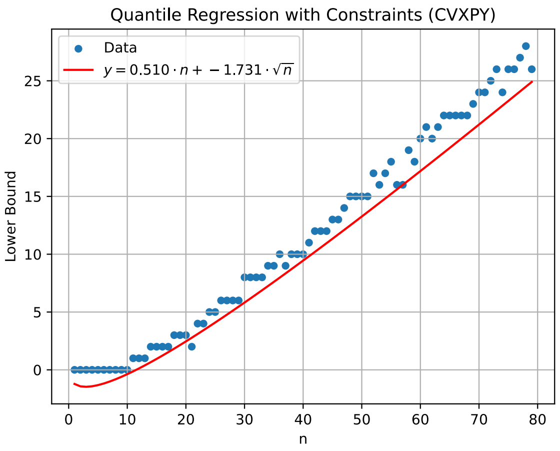

Fig. S3: Lower bound edit distance fit. For each length (x-axis) we performed 1 million (uniform) random DNA versus (uniform) random DNA comparisons, and plotted the lowest observed edit distance (lower bound, y-axis). We then fitted a line through the lower 1% quantile, resulting in $\theta_{\mathrm{emp}}(a) = \max\left(0, \lceil 0.51 \cdot a - 1.7312 \cdot \sqrt{a} \rceil\right)$. The fit was obtained by solving a constrained optimisation problem (minimising the fitting error while enforcing non-negativity) using the Python package cvxpy. Naturally, with more data, the chance of hits in reads increases, and choosing cut-offs will remain a balance between assigned reads and false positives. Nevertheless, the cut-offs from this fit have been shown to work well in practice[7]

[2023]: BC05, 372/393, 94.7%; Di Pilato et al. [2025]: BC45, 61/202, 30.2%). Scanning all untrimmed reads for the fusion pattern revealed that 3.3% of all reads (165,396) contained such a double-barcode fusion.

In the 968 reads where the two barcodes differed (Section 3.2), Dorado consistently reported the outer copy and Barbell the inner copy. To evaluate which assignment was correct, we compared read-level taxonomic annotations with those of the assemblies linked to the assigned barcodes. This approach is limited by the uncertainty of read-level annotations (here filtered at $\geq$ 100 bp hit length; see Section 3.2) and by the fact that nine species were present in two samples (Table S1). Taxonomic annotation supported the inner barcode in 515 cases (53.2%) versus 43 (4.4%) for the outer, with the remainder being unclassified.

F. Pore signal examples

To study the pore signal we used the raw pod5 files and basecalled these using Dorado's super-accurate model, emitting the move table (`--emit-moves`). We then converted the pod5 files to slow5 using blue-crab [Gamaarachchi et al., 2022], and visualised the pore signals and basecalled reads using Squigualiser [Samarakoon et al., 2024], see Figure S8. In case of secondary structure formation we expected the pore signal intensity (y-axis) to increase drastically (e.g. double). However, this was not the case.

G. Analysis of SRA datasets

To investigate the barcode pattern in datasets other than the one sequenced by us, we queried the SRA database for the terms

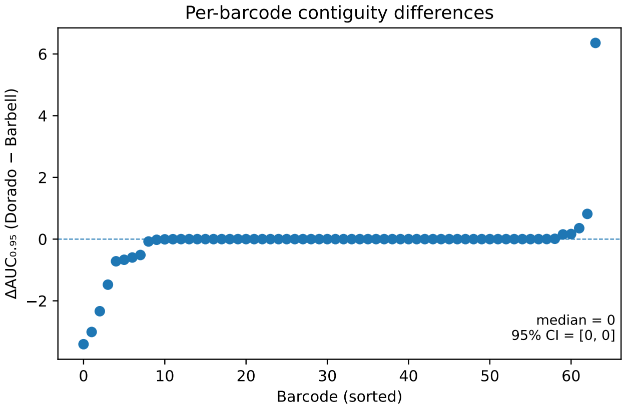

Fig. S4: Per-barcode differences in assembly contiguity between assemblies from Dorado and Barbell trimmed reads. For each assembly, contigs were ordered from longest to shortest and the cumulative assembled length was plotted as a function of contig rank, then normalised by the total assembly length. The area under this curve up to 95% of the assembly (AUC0.95) provides a quantitative summary of assembly contiguity, reflecting how many contigs are required to recover most of the assembly: more contiguous assemblies reach 95% with fewer contigs and therefore have lower AUC0.95 values. Differences between methods are shown as $\Delta\mathrm{AUC0.95} = \mathrm{AUC0.95, Dorado}$ - $\mathrm{AUC0.95, Barbell}$ across 64 shared barcodes. Positive values indicate that Dorado assemblies require more contigs to reach 95% of the assembled sequence (i.e., are more fragmented), whereas negative values indicate greater fragmentation in Barbell assemblies. This metric captures differences in contig length distributions (assembly fragmentation) and does not reflect base-level sequence accuracy. The dashed line denotes $\Delta\mathrm{AUC0.95} = 0$. The median difference was 0 (bootstrap 95% CI: [0, 0]), indicating similar assembly contiguity between the two methods.

"SQK-RBK114" and "SQK-NBD114" and selected 8 random datasets for each (including our own for rapid). These datasets along with the identifiers used in Figure 4 are listed in Table S2.

Read-level re-analyses and trimming

This appendix documents the read-level re-analyses used to validate and repair assemblies that contained barcode or flank sequences. Full command lines, parameters, and logs are provided here for reproducibility.

Photobacterium leiognathi (`CP131573.1`). The uploaded contigs were 3,089,627 bp and 1,431,556 bp. We re-downloaded 89,663 raw reads from SRA. Pattern counts and trimming summary:

- 73,580 reads (82%) contained the expected single-flank pattern `Ftag[fw,*,@left(0..250)]`.
- 1,299 reads (1.4%) contained a double-flank arrangement `Ftag[fw,*,@left(0..250)]_Ftag[fw,*,@prev_left(0..250)]`.

Trimming with Barbell (default options for SQK-RBK114-96) and reassembly produced three circular contigs (3,176,913 bp; 1,497,394 bp; 15,997 bp) and a small linear contig (4,109 bp). The 1.49 Mb contig matched `CP131573.1` without barcode sequences.

E. coli plasmid (`CP165501.1`). We re-downloaded 113,399 raw reads from SRA and demultiplexed with Barbell (default; SQK-RBK114-96):

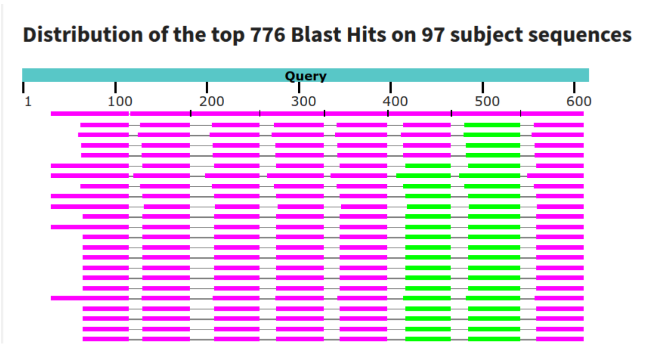

Fig. S5: Artefact read with 8 barcodes. This figure shows the BLAST output for read `52369018-4a3c-433b-881b-e46226500fb6` (611 bp) against all possible rapid barcode flanks and barcodes. The read consists entirely of barcode and flank sequences. Barbell detected 8× an `Ftag` in this read. The "Query" represents the read sequence. Each bar corresponds to a BLAST hit: pink bars indicate alignment scores of 80–200, and green bars 50–80. Because one region can match multiple barcode or flank sequences, matches appear underneath each other, with the highest-scoring ones shown on top. As expected, we observe eight distinct blocks (or "columns"), matching the number of `Ftag`s detected by Barbell.

Table S2. SRA run accessions corresponding to labels D1–D16 in Figure 4.

Label	SRA accession
D1	SRR36128788
D2	SRR36347832
D3	SRR36496803
D4	SRR36760490
D5	SRR36804021
D6	SRR36844237
D7	SRR37153304
D8	SRR37504295
D9	ERR15726106[a]
D10	SRR36318375
D11	SRR36516319
D12	SRR36688800
D13	SRR36911183
D14	SRR37228286
D15	SRR37630668
D16	SRR37857610

[a] Our dataset.

- 97,461 reads (86%) contained the expected single-flank pattern.
- According to Barbell, 76.4% of reads were assigned to BC09 and 13.1% to BC10.

Assembling reads trimmed and binned for BC09 produced a 43,165 bp contig matching `CP165501.1` from positions 111 to 39,256, consistent with removal of barcode overhangs at both contig ends.

Notes and reproducibility. All trimming and assembly commands, parameter files, and run logs (including the exact Barbell and assembler versions used) are archived with the Zenodo record cited in the main text, and code can be found at Zenodo[8].

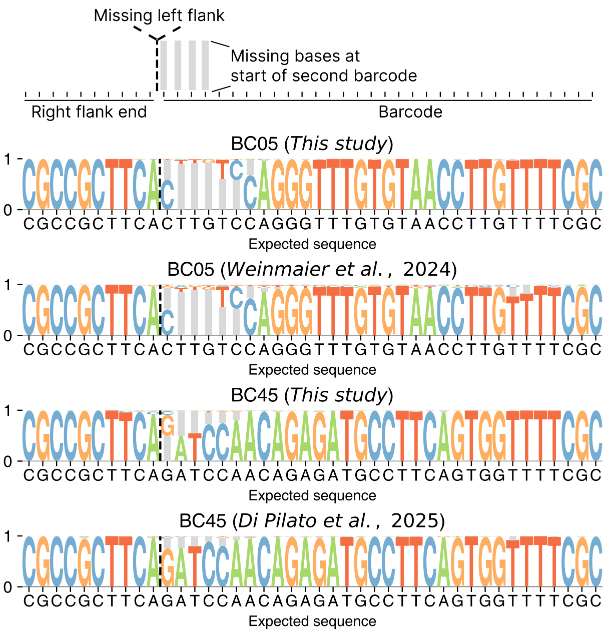

Fig. S6: Detection of fused rapid barcodes and associated deletions. Among 213,016 reads with two left barcodes, 100,042 showed an unusual arrangement in which the right flank adjacent to the first barcode was directly fused to the second barcode (see Appendix A.3). The sequence logos show the fusion junctions for BC05 and BC45 in our and public datasets. Letter height indicates base frequency; grey bars mark alignment gaps (missing bases). In typical reads, a left flank, barcode, and right flank are observed in order, whereas fusion reads show the right flank of the first barcode (ending with TTCA, dashed line) joined directly to the second barcode, always lacking its left flank (not shown) and first part of the second barcode. Deletions within the first 6 bp of the second barcode occurred in 95.4% (BC05) and 52.3% (BC45) of our reads, and with comparable frequencies in public datasets (94.7% and 30.2%, respectively). Loss of the first 1–6 bp of the second barcode was thus more frequent in fusions involving BC05 than BC45.

[8] `https://doi.org/10.5281/zenodo.19494282`

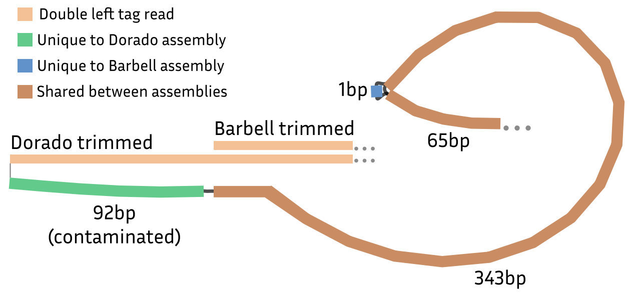

Fig. S7: Merged assembly graphs for a *Saccharomyces cerevisiae* contig. Genome assembly of a single contig from *Saccharomyces cerevisiae* using Dorado- and Barbell-trimmed reads (23,203 bp vs. 23,050 bp). Shown are the first 500 bp of the Dorado assembly and the corresponding region from the Barbell assembly. The nodes represent unitigs, and the edges their connections. The assemblies were identical except for the first 92 bp and a single nucleotide difference. The extra 92 bp in the Dorado assembly originated from a single barcode sequence left untrimmed by Dorado. The difference was caused by one read containing two left barcodes; Dorado removed only the outer barcode and left the inner one intact, since it was incorporated into the assembly. In contrast, Barbell removed both barcodes, preventing this contamination.

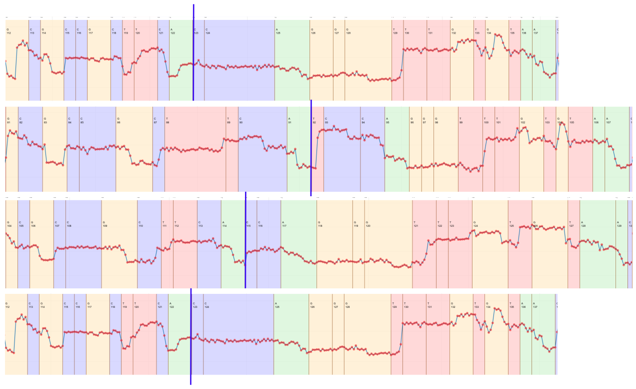

Fig. S8: Examples of the pore signal (line with red dots) for BC05 reads. The vertical blue line indicates the fusion point between the end of the right flank (`..GCTTCA`) and the beginning of the partial BC05 barcode (`CTTGTCCAGGGTTTGTGTAACCTT`). The colours indicate the basecalled bases, G=yellow, C=blue, T=red, A=green. We did not observe abnormally long stretches of signal without any basecalled bases.

Alg. 1: Pseudocode for Barbell's `annotate` step.

Require: β: set of barcode strings; τ_N: tag with masked barcode; R: read sequence
Ensure: T': set of collapsed tag calls

$\triangleright$ — *Parameters* — $\triangleleft$
$S_{\min} \leftarrow 0.2$ $\triangleright$ *Minimum acceptable normalised score*
$S_{\text{diff}} \leftarrow 0.1$ $\triangleright$ *Minimum gap between top and second score*
$W \leftarrow 5$ $\triangleright$ *Extra padding around barcode window*
$S_{\text{perfect}} \leftarrow S_3(\texttt{"24="}, 0.5)$ $\triangleright$ *Ideal perfect score*
$\theta_B \leftarrow 20$ $\triangleright$ *Fixed edit distance cutoff for barcodes*

$\triangleright$ — *Stage 1: Flank detection* — $\triangleleft$
$\theta_\tau \leftarrow \max\left(0, \left\lceil 0.5100 \cdot (\tau_N - |B|) - 1.7312 \cdot \sqrt{\tau_N - |B|} \right\rceil\right)$
$M_\tau \leftarrow \text{Sassy}(\tau_N, R, \theta_\tau)$
$T \leftarrow [\,]$
for $m \in M_\tau$ **do**
  $start \leftarrow m.start$
  $strand = m.strand$
  $mask_{start} \leftarrow \max(0, start + |F_\ell| - W)$
  $mask_{end} \leftarrow \min(|R|, start + |F_\ell| + |B| + W)$
  $\triangleright$ *Note that* $R[i \dots j] := r_i \dots r_{j-1}$ *denotes a right-exclusive substring of* R. $\triangleleft$
  $mask \leftarrow R[mask_{start} \dots mask_{end}]$

  $\triangleright$ — *Stage 2: Barcode matching* — $\triangleleft$
  $matches \leftarrow [\,]$
  $scores \leftarrow [\,]$
  for $B \in \beta$ **do**
    $M_B \leftarrow \text{Sassy}(B, mask, \theta_B)$
    for $m_b \in M_B$ **do**
      if $strand \neq m_b.strand$ **then**
        continue
      $S_{\text{abs}} \leftarrow S_3(m_b.cigar, 0.5)$
      $S_{\text{rel}} \leftarrow S_{\text{abs}}/S_{\text{perfect}}$
      $\text{append}(matches, m_b)$
      $\text{append}(scores, (S_{\text{rel}}, |matches|))$

  $\triangleright$ — *Stage 3: Candidate selection* — $\triangleleft$
  if $scores \neq \emptyset$ **then**
    $scores \leftarrow \text{sort\_desc}(scores)$
    $(s_1, i_1) \leftarrow scores[0]$
    $s_2 \leftarrow \begin{cases} scores[1].S_{\text{rel}}, & |scores| > 1 \\ 0, & \text{otherwise} \end{cases}$
    $\Delta \leftarrow s_1 - s_2$
    if $s_1 \geq S_{\min} \land \Delta \geq S_{\text{diff}}$ **then**
      $\text{append}(T, matches[i_1])$ $\triangleright$ *confident barcode* *Ftag*
    else
      $\text{append}(T, m)$ $\triangleright$ *ambiguous* $\rightarrow$ *store flank (Fflank)*
  else
    $\text{append}(T, m)$ $\triangleright$ *no barcode* $\rightarrow$ *store flank (Fflank)*

$\triangleright$ — *Stage 4: Post-processing* — $\triangleleft$
$\triangleright$ *If overlap (smaller vs. larger match region) bigger than 70%, collapse, prioritising Ftag over Fflank* $\triangleleft$
$T' \leftarrow \text{collapse}(T)$
return T'
